# Response to “*Schistosoma bovis* Infecting Humans in Nigeria”

**DOI:** 10.4269/ajtmh.24-0714a

**Published:** 2025-01-07

**Authors:** Antonia Omenebele Enudi

**Affiliations:** Animal and Environmental Biology Department Faculty of Science Delta State University Abraka Delta State, Nigeria E-mail: ebelenudi@hotmail.com

I would like to clarify our study.

To characterize infections, we used the partial cox1 gene, which has been established to be effective in discriminating *Schistosoma haematobium* from closely related species to identify the causative agent of urinary schistosomiasis in an endemic setting, where humans and livestock share water bodies.

At the time of this study, molecular analysis had not been carried out despite decades of disease transmission. Thus, we pooled samples as a cost-effective way to gain understanding of the molecular epidemiology of the disease using an established protocol.^1^

Our findings suggest backcrossing and introgression.^2^ Although, we did not use a nuclear marker as observed, it is noteworthy that Boissier et al.^3^ observed limitations in the use of the partial cox1 gene and nuclear rDNA ITS region, and that hybrid backcrossed schistosome generations can affect nuclear genetic profiles.

It is our hope that our findings will pave the way for further molecular studies on urinary schistosomiasis, given that advances in genotyping methods should enable improved interpretation of genetic interactions within the *S. haematobium* group.
